# Mediators of the association between educational attainment and type 2 diabetes mellitus: a two-step multivariable Mendelian randomisation study

**DOI:** 10.1007/s00125-022-05705-6

**Published:** 2022-04-28

**Authors:** Jia Zhang, Zekai Chen, Katri Pärna, Sander K. R. van Zon, Harold Snieder, Chris H. L. Thio

**Affiliations:** 1 Department of Epidemiology, University Medical Center Groningen, University of Groningen, Groningen, the Netherlands; 2 Shenzhen Center for Disease Control and Prevention, Shenzhen, Guangdong China; 3 Institute of Genomics, University of Tartu, Tartu, Estonia; 4 Department of Health Sciences, University Medical Center Groningen, University of Groningen, Groningen, the Netherlands

**Keywords:** Educational attainment, Mediation, Mendelian randomisation, Type 2 diabetes

## Abstract

**Aims/hypothesis:**

Type 2 diabetes mellitus is a major health burden disproportionately affecting those with lower educational attainment (EA). We aimed to obtain causal estimates of the association between EA and type 2 diabetes and to quantify mediating effects of known modifiable risk factors.

**Methods:**

We applied two-step, two-sample multivariable Mendelian randomisation (MR) techniques using SNPs as genetic instruments for exposure and mediators, thereby minimising bias due to confounding and reverse causation. We leveraged summary data on genome-wide association studies for EA, proposed mediators (i.e. BMI, blood pressure, smoking, television watching) and type 2 diabetes. The total effect of EA on type 2 diabetes was decomposed into a direct effect and indirect effects through multiple mediators. Additionally, traditional mediation analysis was performed in a subset of the National Health and Nutrition Examination Survey 2013–2014.

**Results:**

EA was inversely associated with type 2 diabetes (OR 0.53 for each 4.2 years of schooling; 95% CI 0.49, 0.56). Individually, the largest contributors were BMI (51.18% mediation; 95% CI 46.39%, 55.98%) and television watching (50.79% mediation; 95% CI 19.42%, 82.15%). Combined, the mediators explained 83.93% (95% CI 70.51%, 96.78%) of the EA–type 2 diabetes association. Traditional analysis yielded smaller effects but showed consistent direction and priority ranking of mediators.

**Conclusions/interpretation:**

These results support a potentially causal protective effect of EA against type 2 diabetes, with considerable mediation by a number of modifiable risk factors. Interventions on these factors thus have the potential of substantially reducing the burden of type 2 diabetes attributable to low EA.

**Graphical abstract:**

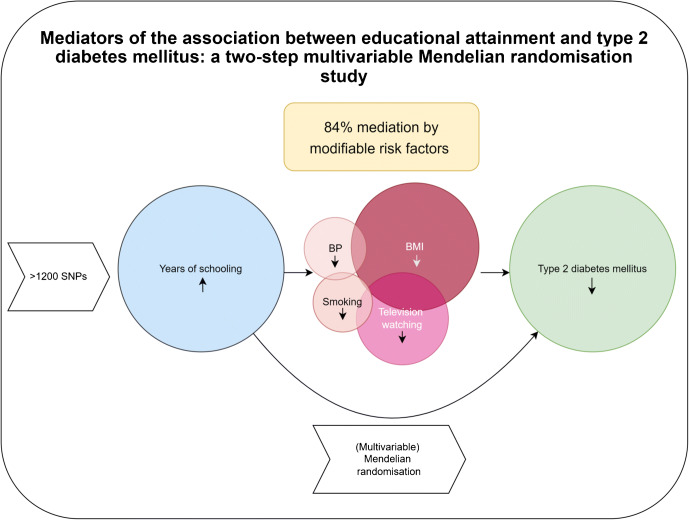

**Supplementary Information:**

The online version contains peer-reviewed but unedited supplementary material available at 10.1007/s00125-022-05705-6.

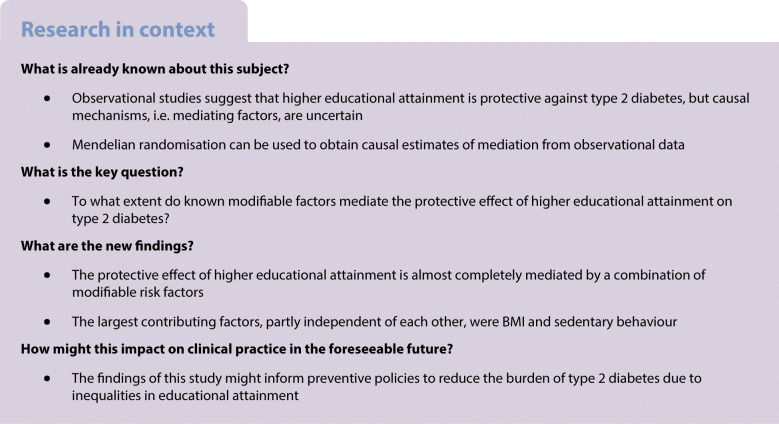



## Introduction

Type 2 diabetes is a multifactorial group of disorders in which impaired insulin secretion and/or insulin resistance results in dysregulated carbohydrate, lipid and protein metabolism [1]. It confers an increase in risk of cardiovascular disease and all-cause mortality [2, 3]. Global prevalence has been continuously rising over the past few decades, with type 2 diabetes projected to affect 9.9% of the world population by the year 2045 [4–8], thus posing an increasingly unsustainable global health burden [9].

A large and consistent body of epidemiological data suggests that those with lower educational attainment (EA) are disproportionately affected by type 2 diabetes [10]. This association is likely mediated by modifiable risk factors, such as obesity, sedentary behaviour, physical activity (PA), smoking and blood pressure [11–15]. Knowledge of mediation in the EA–type 2 diabetes association will inform public health policies, e.g. by prioritising targets for intervention to reduce the excess risk of type 2 diabetes due to low EA. Our current knowledge of mediating pathways is predominantly based on traditional observational studies that are sensitive to confounding and reverse causation. Therefore, it is uncertain to what extent the associations between EA and type 2 diabetes, and their intermediates, are confounded or affected by reverse causation.

A well-acknowledged method to support causal inference in observational data is Mendelian randomisation (MR). This method uses SNPs, identified in genome-wide association studies (GWAS) to be strongly associated with an exposure, as instrumental variables [16]. Under a number of assumptions, MR yields estimates of an exposure–outcome relation that are less likely to be biased due to unobserved confounding. Recent advances in MR methodology include multivariable MR (MVMR), which can be applied to investigate mediation [17].

Previous MR studies have provided support for a potential causal effect of EA (measured by years of schooling) on coronary artery disease [18], with evidence of mediation through risk factors such as BMI, smoking and blood pressure [19]. For type 2 diabetes risk, recent MR studies have also provided evidence of such a causal effect of EA [20–22]. However, these studies did not assess mediation by modifiable factors [21, 22], while one recent study only examined mediation by BMI and smoking [23]. Furthermore, some MR studies relied on genetic associations leveraged from less recent, less precise GWAS data [20, 21]. Recent GWAS on EA [24] and type 2 diabetes [25] have yielded more precise estimates of SNP effects due to their larger sample size compared with less recent GWAS. Updating the results from previous MR studies on EA and type 2 diabetes, as well as assessing potential mediation, using the most recent GWAS data would result in more precise insights into the causal structure underlying the EA–type 2 diabetes association.

We therefore aimed to obtain causal estimates of the association between EA and type 2 diabetes and to characterise the causal structure by assessing mediation effects of BMI, sedentary behaviour, PA, smoking and blood pressure in an MVMR framework. In addition, we aimed to obtain observational mediation estimates from the 2013–2014 National Health and Nutrition Examination Survey (NHANES).

## Methods

### Overall study design

This study used a two-step MR analysis of genetic summary data to investigate to what extent BMI, blood pressure, smoking, sedentary behaviour (daily hours of television watching) and PA explain the protective effect of EA on type 2 diabetes risk. In addition, we estimated mediation in data from the NHANES 2013–2014 using traditional observational mediation analysis techniques.

### Outcome definitions

In the original GWAS [24], educational level was categorised according to the International Standard Classification of Education (ISCED) 2011 [26], converted to US years of schooling and standardised, with each unit representing 4.2 years of schooling. Type 2 diabetes in the original GWAS was defined by diagnostic fasting glucose, casual glucose, 2 h plasma glucose or HbA_1c_ levels; use of glucose-lowering medication (by Anatomical Therapeutic Chemical code or self-report); or type 2 diabetes history from electronic medical records, self-report and varying combinations of each, depending on the contributing cohort [25]. Mediator selection was based on potential for modification, observational epidemiological evidence of mediation in the EA–type 2 diabetes relation and availability of comprehensive GWAS data. BMI was calculated by dividing weight (kg) by height squared (m^2^) [27]. Sedentary behaviour was measured by daily hours of television watching [28]. Systolic blood pressure (SBP) and diastolic blood pressure (DBP) values were the mean of two automated or two manual blood pressure measurements [29]. Smoking behaviour was a binary trait: participants who reported ever being a regular smoker in their life were defined as smokers; the remaining participants were defined as non-smokers [30]. PA was measured by accelerometry and reported as SDs of metabolic equivalent of task [31].

### MR methods

For a brief discussion of assumptions underlying MR, see electronic supplementary material (ESM) Methods 1. We used two-sample MR (2SMR) methods using GWAS summary level data [32]. Two-step 2SMR (ESM Fig. 1) was used to assess whether an intermediate trait has a mediation effect between exposure and outcome [33]. The first step was to estimate the causal effect of EA on potential mediators using SNPs to genetically predict years of schooling. In the second step, SNPs for the potential mediating risk factors were used to genetically predict these mediators and to estimate their causal effect on the outcome, adjusting for EA using MVMR. The total effect of EA was then decomposed into a direct effect (i.e. the effect of EA on type 2 diabetes independent of the mediator) and an indirect effect (i.e. the effect of EA on type 2 diabetes via the mediator). This approach is currently being widely applied [19, 34].

For these analyses, we obtained summary statistics of the genetic associations from the most recent GWAS for each respective phenotype. Table 1 summarises the GWAS data used in this study.

**Table 1:** Overview of GWAS data used in MVMR

Phenotype	Unit	Number of participants	Number of lead SNPs	Explained variance by lead SNPs	Ancestry	Consortium/cohort	Author	Year of publication	PubMed ID
Years of schooling [24]	SD (4.2 years)	1,131,881	1271	11%	European	SSGAC	Lee et al	2018	30,038,396
BMI [27]	kg/m^2^	681,275	941	6%	European	GIANT	Yengo et al	2018	30,124,842
SBP [29]	mmHg	775,601	970	5.7%	European	UKBB+ICBP	Evangelou et al	2018	30,224,653
DBP [29]	mmHg	775,601	962	5.3%	European	UKBB+ICBP	Evangelou et al	2018	30,224,653
Type 2 diabetes [25]	Yes vs no	74,124 cases 824,006 control participants	403	18%	European	DIAGRAM	Mahajan et al	2018	30,297,969
Smoking initiation [30]	Ever vs never	557,337 cases 674,754 control participants	378	2.3%	European	GSCAN	Liu et al	2019	30,643,251
PA (accelerometry) [31]	SD (not further specified)	91,105	5	0.06%	European	UKBB	Doherty et al	2018	30,531,941
TV watching [28]	SD (1.5 h)	408,815	152	2.3%	European	UKBB	van de Vegte et al	2020	32,317,632

DIAGRAM, DIAbetes Genetics Replication And Meta-analysis; GIANT, Genetic Investigation of Anthropometric Traits; GSCAN, GWAS & Sequencing Consortium of Alcohol and Nicotine use; ICBP, International Consortium for Blood Pressure; SSGAC, Social Science Genetic Association Consortium; TV, television; UKBB, UK Biobank

#### Instrument selection

All selected SNPs and their associations with EA, mediators and type 2 diabetes were extracted from the GWAS studies in Table 1. For EA, genetic instruments were selected from the Social Science Genetic Association Consortium GWAS meta-analysis of years of schooling in 1,131,881 individuals of European ancestry [24]. A total of 1271 independent (*r*^2^ < 0.1) genome-wide significant (*p* < 5×10^−8^) SNPs were used as the primary genetic instruments.

For each mediator, genetic instruments were selected from the most recent large-scale GWAS data. We then selected genome-wide significant SNPs for each trait (*p* < 5×10^−8^ for SBP/DBP, television watching, smoking and PA; *p* < 1×10^−8^ for BMI). We applied pairwise linkage disequilibrium (LD) thresholds from the original GWAS for each mediating trait, with SNPs for each trait adhering to an LD cut-off of *r*^2^ < 0.1 within a window of 1 MB, except for television watching (LD *r*^2^ < 0.005 within 5 MB) and smoking (LD *r*^2^ < 0.1 within 500 kB). Then, for all SNPs, we harmonised coding and non-coding alleles in the summary statistics of each GWAS. In case of palindromic SNPs, we inferred strand based on allele frequency. Palindromic SNPs with ambiguous allele frequency (frequency 0.3–0.7) were removed.

#### Effect of EA on type 2 diabetes

We obtained estimates of the total effect of EA on type 2 diabetes using straightforward 2SMR. Here, single SNP estimates of the effect of EA on type 2 diabetes were investigated by calculating Wald ratios with standard errors derived using the delta method. We used inverse variance-weighted meta-analysis to pool Wald ratios as our main method [32].

#### Mediation analysis

We used inverse variance weighting (IVW) as our main approach to estimate the effect of EA on each mediator. We used regression-based MVMR to estimate the effect of each mediator on type 2 diabetes risk while adjusting for the genetic effect of the instruments on EA [35]. For the individual mediation effect of each risk factor (BMI, SBP, DBP, smoking, PA and television watching), we used the product of coefficients method as our main method to estimate the indirect effect (that is, the effect of EA on type 2 diabetes through the mediator) [36]. This involved first estimating the effect of EA on each mediator individually, then multiplying the EA–mediator effect with the EA-adjusted effect of the mediator on the outcome [34]. The proportion of the total effect of EA on type 2 diabetes that was mediated by each risk factor separately was estimated by dividing the indirect effect by the total effect. Standard errors were derived by using the delta method, using effect estimates obtained from 2SMR analysis.

To estimate the combined proportion mediated, we used the difference in regression coefficient MVMR approach to adjust the genetic effect for several mediators simultaneously (such as BMI + SBP) to obtain the direct effect of EA on type 2 diabetes. Then, the combined indirect effect of considered mediators was the residual of the total effect. We explored all possible meaningful combinations of mediators to find the combination of mediation with the highest proportion mediated and to assess potential overlap between mediators.

Although PA was initially included in our selection of potential mediating risk factors, GWAS data on this variable were limited with regard to power and number of available genetic instruments: only two SNPs were available, precluding meaningful mediation analysis using the product of coefficients method. We therefore decided to abandon further investigation of PA as an intermediate trait in both our main MR and observational analyses, although we were able to examine mediation by PA using the difference method.

#### MR sensitivity analyses

We conducted several sensitivity analyses to evaluate the robustness of the MR results. First, in addition to our primary IVW meta-analysis, we applied 2SMR methods that are robust to violations of the assumptions regarding horizontal pleiotropy of SNPs (ESM Methods 1). Second, to validate estimates of the product of coefficients method, we used the MVMR approach to estimate the individual mediation effect of each mediator using the difference method. In this approach, the indirect effect of each mediator was estimated by subtracting the direct effect of education from the total effect. Third, we used the MVMR-Egger method to examine the robustness of the MVMR-IVW results. Fourth, to assess the validity of our mediation model we conducted reverse MR using genetic instruments for each purported mediator to explore bidirectionality between EA and potential mediators.

All MR analyses were conducted using R (version 4.0.2) [37] and the *TwoSampleMR* R package version 0.5.6 [38].

### Observational mediation analysis in NHANES

Details on the sample and methods of the observational mediation analysis can be found in ESM Methods 2. Briefly, we applied standard regression-based mediation analysis methods to explore mediation through pathways presented in ESM Fig. 2.

## Results

### MR analysis

#### Genetic instruments

Detailed information on SNPs and their associations with EA, mediators and type 2 diabetes can be found in the ESM Data of SNPs.

#### Effect of EA on type 2 diabetes

Each SD (4.2 years of schooling) higher genetically predicted EA was associated with 0.53 times lower odds of type 2 diabetes (OR 0.53; 95% CI 0.49, 0.56).

#### Effect of EA on mediators

**Fig. 1:**
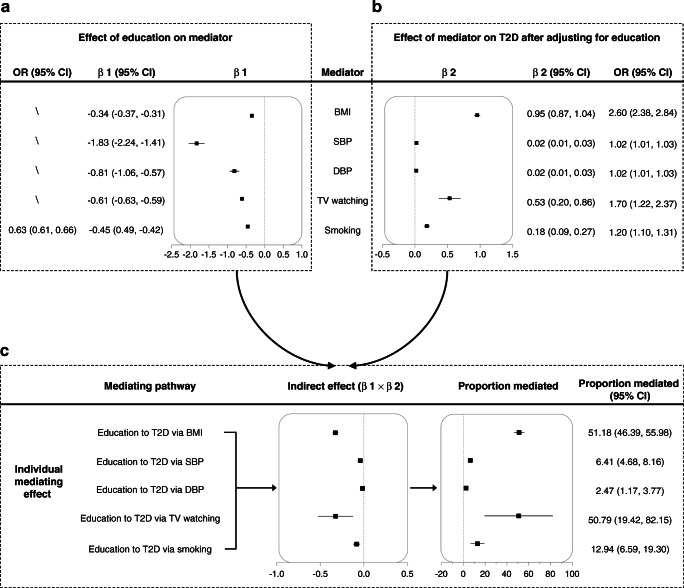
(**a**) MR-estimated effects of EA (per 4.2 years of schooling) on each mediator separately, presented as β/OR with 95% CI. (**b**) MR-estimated effects of each mediator separately on type 2 diabetes after MVMR adjustment for education, presented as β/OR with 95% CI. (**c**) MR-estimated effects of indirect effects of each mediator separately, by product of coefficients method with delta method-estimated 95% CIs. MR-estimated proportions mediated (%) are presented with 95% CIs. T2D, type 2 diabetes; TV, television

Effects of genetically predicted EA on each mediator are shown in Fig. 1a. Each SD (4.2 years of schooling) higher genetically predicted EA was associated with lower BMI (β = −0.34 kg/m^2^; 95% CI −0.37, −0.31); less television watching (β = −0.61 SD of television watching; 95% CI −0.63, −0.59, translating to 0.92 h less television watching per 4.2 years of schooling); lower odds of smoking (OR 0.63; 95% CI 0.61, 0.66); lower SBP (−1.83 mmHg; 95% CI −2.24, −1.41); lower DBP (β = −0.81 mmHg; 95% CI −1.06, −0.57); and more PA (β = 0.08 SD; 95% CI 0.05, 0.12).

#### Effect of mediators on type 2 diabetes with adjustment for EA

Figure 1b shows that each mediator was significantly associated with type 2 diabetes after adjusting for EA. We excluded PA from our analysis as only two out of five PA SNPs could be used in MVMR, precluding meaningful analysis using the product of coefficients method. A 1 kg/m^2^ higher genetically predicted BMI was associated with 2.60 times higher odds of type 2 diabetes (95% CI 2.38, 2.84). A 1 mmHg higher genetically predicted blood pressure (SBP/DBP) was associated with higher odds of type 2 diabetes (OR 1.02; 95% CI 1.01, 1.03). One SD (1.5 h) longer genetically predicted television watching was associated with 1.70 times higher odds of type 2 diabetes (95% CI 1.22, 2.37). Compared with never-smokers, the odds of type 2 diabetes among genetically predicted ever-smokers were 1.20 times higher (95% CI 1.10, 1.31).

#### Individual proportion mediated

Figure 1c displays the proportion of the effect of EA on type 2 diabetes explained by each mediator separately. BMI explained 51.18% (95% CI 46.39%, 55.98%) of the total effect of EA on type 2 diabetes, while television watching explained 50.79% (95% CI 19.42%, 82.15%). Smoking explained 12.94% (95% CI 6.59%, 19.30%) of the total effect. DBP and SBP only subtly mediated the total effect of EA (2.47% for DBP, 6.41% for SBP).

#### Combined proportion mediated

**Fig. 2:**
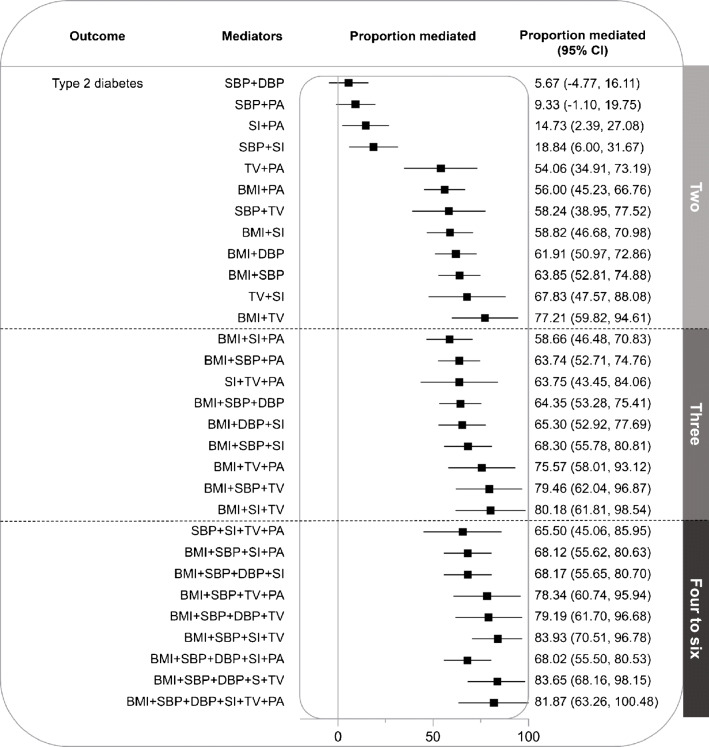
MR estimates of combined proportions mediated by multiple mediators, presented as percentages with 95% CIs. SI, smoking initiation (ever vs never); TV, television watching

We examined the proportion mediated of different combinations of mediating variables. This was done in an effort to find the combination that explained the most variation in the EA–type 2 diabetes association, as well as to investigate potential overlap in effects between mediators (Fig. 2).

Combining any one of the other mediators with BMI was observed to increase the mediating proportion to around 60–77%. The same trend could be observed when combining television watching with any of the other mediating variables.

Among the two-mediator combinations, the combination of BMI + SBP mediated 63.85% (95% CI 52.81%, 74.88%) of the effect of EA on type 2 diabetes. The BMI + television watching combination showed the highest proportion mediated (77.21%; 95% CI 59.82%, 94.61%). This relatively large combined mediation effect suggests little overlap between BMI and television watching.

Among the three-mediator combinations, the combination of BMI + television watching + smoking accounted for the largest mediation effect (80.18%; 95% CI 61.81%, 98.54%).

As expected, any combination of both SBP and DBP did not result in a meaningful increase in proportion mediated compared with a combination with either SBP or DBP.

The four-mediator combination with the largest proportion mediated was BMI + SBP + smoking + television watching, accounting for 83.93% (95% CI 70.51%, 96.78%) of the effect of EA.

No meaningful increase in proportion mediated was observed for five- and six-mediator combinations compared with that of the most effective four-mediator group.

#### MR sensitivity analyses

We assessed heterogeneity using Cochran’s *Q* statistic. We observed substantial heterogeneity from genetic instruments for EA to the outcome and mediators (ESM Table 1), indicating potential pleiotropy of SNP effects. To evaluate potential directional horizontal pleiotropy, we performed MR-Egger regression to assess whether the mean value of the Egger intercept was non-zero, in which case pleiotropy could be directional [39]. In our study, we found no significant directional pleiotropy for any of the 2SMR analyses (*p* > 0.05, ESM Table 2). In addition, MR-weighted median methods were generally consistent with MR-IVW with regard to magnitude and direction (ESM Tables 3, 4, ESM Fig. 3), suggesting that any horizontal pleiotropy did not greatly bias our results.

To assess the consistency of our main MR product of coefficients estimates of individual mediation, we performed additional MVMR mediation analysis using the difference in regression coefficient method; estimates were highly similar (ESM Fig. 4). In contrast to the product of coefficients method, we were able to examine mediation effects of PA using the difference method; here, PA explained 4.47% (95% CI 0.69%, 8.31%) of the total effect (ESM Fig. 4). The combined proportion mediated of PA and BMI was not larger than that of BMI alone, suggesting substantial overlap in mediation effects between the two (ESM Fig. 4, Fig. 2). Results from MVMR-Egger sensitivity analyses were highly consistent with those from MVMR-IVW analysis (ESM Table 5), suggesting low risk of bias due to horizontal pleiotropy. There was reasonable instrument strength (*F* > 10) of SNPs for EA, BMI, SBP and DBP in all MVMR analyses. However, conditional instrument strength for television watching, smoking initiation and PA was low.

In reverse MR analyses, higher BMI suggestively reduced EA, although this reverse effect was likely driven by horizontal pleiotropy (*p*_Egger intercept_ = 0.0166). There was no evidence for a causal effect of SBP, DBP and PA on EA. Reverse MR suggested that television watching and smoking reduce EA level (ESM Table 6), indicating that some bidirectionality exists between these factors and EA, possibly affecting validity of our mediation model. Therefore, we constructed a final mediation model including only BMI, SBP and DBP, while excluding television watching and smoking. In this mediation model, the combined proportion explained by BMI, SBP and DBP was 64.35% (95% CI 53.28%, 75.41%) (ESM Figs. 5, 6).

### Observational mediation results in NHANES

Descriptive statistics of 1912 participants from the NHANES 2013–2014 are presented in ESM Table 7. In observational analysis (ESM Table 8), each 4.2 year higher EA was associated with lower odds of type 2 diabetes (OR 0.64; 95% CI 0.56, 0.73), similar in magnitude and direction to the MR estimates. Higher EA was associated with lower BMI, less television watching, lower odds of smoking, lower SBP and lower DBP. Product of coefficients estimates of individual proportion mediated were 25%, 16%, 7%, 4% and 0%, respectively, while the total combined proportion mediated estimated by the difference method was 30%.

## Discussion

In this two-step MVMR study, we found evidence suggestive of a causal, protective effect of EA on type 2 diabetes, with up to 84% mediation by a combination of the modifiable factors BMI, television watching, blood pressure and smoking. Observational mediation estimates in the NHANES 2013–2014 were consistent with the MR mediation estimates with regard to directionality and priority ranking of mediators, but overall suggested less pronounced mediation by the risk factors of interest.

In the present study, the MR-estimated causal effect of a 1 SD (4.2 years of schooling) increase in EA was a 47% reduction in odds of type 2 diabetes (OR 0.53, similar to previous MR studies, ORs ranging from 0.39 to 0.61) [20–23].

Previous observational studies of the EA–type 2 diabetes association reported 31–53% mediation by a range of risk factors [12, 13]. A previous MR study found that 64% of the association between EA and type 2 diabetes was mediated by BMI and smoking [23], similar to the combined mediation estimate of BMI and smoking in the present study (58%). In general, observational estimates of mediation are lower than those derived from MR. This could be due to underestimation of associations in observational studies due to confounding or measurement error. Although MR is less sensitive to confounding or measurement error, it has been suggested to yield higher associations given that SNP effects represent an estimate of lifetime exposure [19].

Up to ~84% of the EA–type 2 diabetes association was mediated by traditional (i.e. clinical) risk factors, while ~16% remains unexplained. Potential factors that may explain the remainder of the association include factors such as area deprivation, income, diet, health literacy, healthcare access and psychosocial factors. Many of these factors may be not heritable and therefore not suitable for GWAS and consequently unsuitable for 2SMR. However, these factors are expected to show high overlap with factors investigated in the present study, i.e. BMI, television watching, smoking and blood pressure; we therefore expect that these omitted factors would not have contributed substantially to explaining the EA–type 2 diabetes relation. They might however play a role in intervention strategies to reduce type 2 diabetes risk, e.g. reducing BMI through improving diet and health literacy.

Estimates generated from MR are generally insensitive to reverse causation due to the random assignment of alleles at conception. However, our results suggested a bidirectional negative relationship of television watching and smoking with EA, which may imply that EA could also be a mediator of these two traits, complicating the hypothesised model. Additionally, given the low instrument strength for these two traits, results for these two factors should be cautiously interpreted.

Directly intervening on EA by raising the school-leaving age has been shown to be effective in improving adult health (including type 2 diabetes) and reducing mortality in the UK [40]. Other interventions may involve improving access to education, and improving quality of (health) education. However, such interventions are impractical short-term solutions to reducing the burden of type 2 diabetes. In the present study, we provide evidence of substantial mediation of the EA–type 2 diabetes relation through several risk factors that are more easily modifiable than EA. Although population-wide intervention strategies on these modifiable mediators are expected to increase public health, such an approach may widen the inequality gap of type 2 diabetes risk [41]; a high-risk prevention approach (i.e. interventions that target mediators in those with low EA) may therefore be necessary to reduce socioeconomic disparities in type 2 diabetes risk. Our results ranked BMI and television watching to be the strongest contributing factors in the EA–type 2 diabetes association, interestingly, with relatively little overlap, thus suggesting partly independent effects. Interventions on BMI may involve addressing the obesogenic environment associated with low-socioeconomic status neighbourhoods [42, 43], e.g. by limiting fast food outlets in these neighbourhoods. Screen time interventions (television or otherwise) have previously been successfully implemented to improve diet, weight and PA in children [44]. Future studies may further investigate the feasibility and potential impact of such interventions on adult type 2 diabetes risk.

The present MR study yields population-averaged causal estimates of association and mediation. Given the sex differences in both EA [45] and type 2 diabetes [46], it is likely that associations and mediators are also different between sexes, as shown previously [11]. Future MR studies may investigate this using sex-stratified GWAS data.

The predominance of GWAS, including those used in the present study, were performed in white European ancestry populations from high-income countries; generalisation to other ethnicities and low- and medium-income countries is therefore uncertain. Furthermore, a strong relation exists among ethnicity/race, socioeconomic status and health [47] in multi-ethnic communities. Future studies (including GWAS and MR) should therefore be more inclusive with regard to non-white community dwellers.

Strengths of the present study include that it uses SNPs as genetic instruments to minimise bias due to confounding and reverse causation. We used the most recent large-scale GWAS data to generate highly precise SNP effect estimates, facilitating precise MR analysis. The mediated effects estimated were consistent across the two MR mediation approaches and in the statistical sensitivity analyses. Furthermore, MR estimates were corroborated by observational mediation analyses in NHANES 2013–2014, allowing for triangulation [48] and thus improving the robustness of our findings.

Several limitations must be addressed. First, MR may be biased by pleiotropic effects of SNPs, i.e. genetic variants directly influencing both exposure and outcome: a violation of the exclusion restriction criterion. While sensitivity analyses (i.e. MR-Egger, weighted median) robust to pleiotropy [49, 50] showed consistent results, we did not adjust for cognitive ability, which is highly related to EA and a potential confounder in any EA–outcome relation. However, a recent study showed that MVMR adjustment of EA for cognitive ability did not meaningfully affect MR estimates [22]. Second, a potential limitation of using genetic data on social traits such as EA is that ‘population phenomena’ play a role. These phenomena include population stratification, dynastic effects (i.e. transgenerational effects of non-inherited parental SNP alleles) and assortative mating (e.g. non-random mating based on educational level). Whereas population stratification is usually accounted for in GWAS, dynastic effects and assortative mating are not; SNP–EA associations might thus be confounded and therefore may bias MR estimates [51–54]. Future MR studies might exploit within-family genetic data (e.g. parent–offspring trios, siblings) that have the potential of accounting for such phenomena [55]. Third, the present study assumes absence of exposure × mediator interaction, which currently cannot be modelled in the present 2SMR setting. Fourth, type 2 diabetes and smoking were binary traits, requiring the use of log-odds (as per the original GWAS) in MR analysis for estimating direct and indirect effects. This is non-ideal as ORs are non-collapsible, i.e. marginal ORs are not directly comparable with conditional ORs [56]. Fifth, SNP effects on blood pressure traits were adjusted for BMI in the original GWAS, which subjects MR estimates involving SBP or DBP to potential bias with unpredictable direction [57]. Sixth, sample overlap between GWAS studies may have biased MR estimates towards observational association estimates [58].

To conclude, these results support a potentially causal protective effect of higher EA against type 2 diabetes, with substantial mediation by the modifiable risk factors BMI, television watching and, to a lesser extent, smoking, SBP and DBP. Interventions on these factors thus have the potential of substantially reducing the burden of type 2 diabetes attributable to low EA.

## Supplementary Information

ESM 1 (PDF 511 kb)

ESM 2 (XLSX 506 kb)

## Data Availability

GWAS data are available through the MRC IEU Open GWAS database (https://gwas.mrcieu.ac.uk/). NHANES data are publicly available through the Center for Disease Control (https://wwwn.cdc.gov/nchs/nhanes/). Code can be shared upon request.

## Abbreviations

DBP: Diastolic blood pressure
EA: Educational attainment
GWAS: Genome-wide association studies
IVW: Inverse variance weighting
LD: Linkage disequilibrium
MR: Mendelian randomisation
MVMR: Multivariable MR
NHANES: National Health and Nutrition Examination Survey
PA: Physical activity
SBP: Systolic blood pressure
2SMR: Two-sample MR
