# Supplementary material for: Mediators of the association between educational attainment and type 2 diabetes mellitus: a two-step multivariable Mendelian randomisation study
Source: Diabetologia. 2022 Apr 28;65(8):1364–74. doi: 10.1007/s00125-022-05705-6 (PMC9283137; doi:10.1007/s00125-022-05705-6)
Supplement: Supplementary file 1 — (PDF 511 kb) [file 125_2022_5705_MOESM1_ESM.pdf]

Mediators of the association between educational attainment and type 2 diabetes mellitus: a two-step multivariable Mendelian randomisation study

Electronic supplementary material (ESM)

ESM Methods.
   ESM Methods 1. Mendelian randomisation (MR) assumptions and methods
   ESM Methods 2. Observational mediation analysis in NHANES 2013-2014
ESM SNP data (.xlsx)
ESM Table 1. MR heterogeneity tests
ESM Table 2. MR directional pleiotropy tests
ESM Table 3. MR of EA on T2D
ESM Table 4. MR of EA on mediators
ESM Table 5. Sensitivity analysis of MVMR
ESM Table 6. Reverse MR of mediators on EA
ESM Table 7. Characteristics of 1912 Non-Hispanic white participants from the NHANES 2013-2014 survey
ESM Table 8. NHANES observational estimates of single mediation
ESM Figure 1. Directed acyclic graph for 2-step Mendelian randomisation
ESM Figure 2. Directed acyclic graph of tested pathways in NHANES 2013-2014
ESM Figure 3a. Mendelian randomisation scatterplots of education to type 2 diabetes and mediators associations
ESM Figure 3b. Mendelian randomisation scatterplots of mediators to education associations
ESM Figure 4. MVMR estimates of proportion mediated by each mediator separately using the difference method
ESM Figure 5. Estimates for the effect of education on type 2 diabetes explained by mediators individually (product of coefficients method)
ESM Figure 6. MVMR estimates of combined proportion mediated by selected combinations of mediators

Supplementary References

ESM Methods

ESM Methods 1. Mendelian randomisation assumptions and methods

Causal inference in traditional observational epidemiological studies is limited due to confounding and reverse causation. MR is a method that can be used to uncover causal relationships between exposure and outcome in the presence of such limitations[1]. MR uses SNPs to genetically predict exposures. MR estimates are unconfounded, and thus valid estimates of causality, under a number of key assumptions. The first is the relevance assumption, which assumes that the genetic instruments are strongly associated with the exposure; this assumption is satisfied by the selection of SNPs with robust genome-wide significant ($P<5x10^{-8}$) and replicated associations. The second is the independence assumption, which requires that genetic instruments are not associated with any confounder of the relationship between exposure and outcome; this is assumed to be true due to Mendel's law of independent segregation, in which genetic variants for a certain trait are inherited independently of other traits. The third is the exclusion restriction criterion, which requires that any effect of the genetic instrument on the outcome variable is solely through the exposure variable. This assumption might be violated due to horizontal pleiotropy, in which a genetic instrument might have direct effects on both the exposure and the outcome. To assess robustness of our results against horizontal pleiotropy, we performed sensitivity analyses. Firstly, we assessed the heterogeneity of Wald ratios (i.e. single SNP MR effect estimates) to find evidence of potential pleiotropy; large heterogeneity in Wald ratios is suggestive of horizontal pleiotropy (**Supp Table S1**). Second, we examined MR funnel plots of Wald ratios; assymetry in the funnel plots is suggestive of directional horizontal pleiotropy (data not shown). Third, we examined the Egger intercept; significant deviation from a zero intercept is suggestive of directional horizontal pleitropy (**Supp Table S2**). Finally, we conducted two sensitivity MR analyses that relax the exclusion restriction criterion: a) Mendelian randomisation-Egger (MR-Egger)[2, 3], which allows for estimation of causal effects in the presence of directional horizontal pleitropy, but assumes that the SNP strength is independent of the direct SNP effect on the outcome (InSIDE

assumption[2]); and b) weighted median MR[4], which is based on the median Wald estimate and allows for consistent estimation even when up to 50% of the information comes from invalid, pleiotropic SNPs. Additional assumptions for the two-sample setting of the present study include that sample should represent the same underlying population (in the present study, a European ancestry population) with minimal sample overlap between studies.

ESM Methods 2. Observational mediation analysis in NHANES 2013-2014

We used publicly available data from NHANES 2013-2014. Of this survey, a subset was selected of 1912 non-Hispanic white participants aged 30 years or older, with complete data on age, sex, BMI, hours of TV watching, smoking, SBP and DBP. Educational level was categorized according to ISCED and then assigned a value of years of schooling. Years of schooling was then rescaled so that one unit increase represented 4.2 years of schooling. Participants were categorized as diabetic in case of a HbA_{1c} ≥ 6.5%, fasting plasma glucose ≥ 7 mmol/L, or a 2 hour plasma glucose ≥ 7.8 mmol/L after an oral glucose tolerance test. BMI was calculated by dividing weight (in kg) by height squared (m^2). Television (TV) watching was an ordinal variable but was analyzed as it were a numerical (ranging 0-5 hours) to reflect the MR analysis. For practical and computational reasons, smoking was considered a numerical variable, recoded into ever versus never smoking. SBP and DBP were averaged from multiple repeat blood pressure measurements according to recommendations. Physical activity, as measured by accelerometry, was available in the NHANES 2013-2014 survey, but was left out of the analysis for consistency with the final MR analyses.

We accounted for the complex sampling strategy in NHANES by applying the recommended weighting procedure using the *survey* R-package[5]. We then used the *svyglm*() function implemented in the *survey* R-package for multivariable regression modeling of exposure-mediator-outcome associations. In single mediator models, indirect effects through the mediator of interest were estimated using the product of coefficients method, where coefficients were obtained from linear and logistic regression where appropriate. Standard errors were approximated using the delta method. To estimate proportion mediated by multiple mediators, we applied the difference method. All observational analyses were performed using R version 4.03 software[6].

ESM Table 1a . MR heterogeneity test (MR-Egger)

Exposure	Outcome	N of SNPs	Q statistic	Q df	Q *p*-value
	Type 2 diabetes	1208	3669.07	1206	2.66E-246
	BMI	1202	9531.31	1200	5.32E-82
	SBP	1184	6265.94	1182	4.79E-119
EA	DBP	1188	6723.75	1186	7.34E-99
	TV watching	1211	3337.28	1209	3.58E-198
	Physical activity	1207	2198.20	1205	8.38E-61
	Smoking	1196	3670.22	1194	2.19E-249

ESM Table 1b . MR heterogeneity test (IVW)

Exposure	Outcome	N of SNPs	Q statistic	Q df	Q *p*-value
	Type 2 diabetes	1208	3675.03	1207	6.28E-247
	BMI	1202	9537.97	1201	4.34E-76
	SBP	1184	6266.83	1183	7.13E-121
EA	DBP	1188	6724.90	1187	6.36E-103
	TV watching	1211	3337.60	1210	3.58E-198
	Physical activity	1207	2200.83	1206	6.24E-61
	Smoking	1196	3671.48	1195	2.51E-249

ESM Table 2. MR directional pleiotropy test

Exposure	Outcome	Egger intercept	SE	*p* value
	Type 2 diabetes	-0.0019	0.0013	0.1620
	BMI	-0.0006	0.0006	0.3603
	SBP	-0.0035	0.0086	0.6814
EA	DBP	-0.0023	0.0051	0.6531
	TV watching	0.0001	0.0004	0.7314
	Physical activity	0.0009	0.0007	0.2299
	Smoking	-0.0005	0.0007	0.5218

Results from MR directional pleiotropy tests in the association between Educational attainment and outcomes.

ESM Table 3. Mendelian randomisation analysis of the effect of educational attainment on type 2 diabetes

Exposure	Outcome	Method	N of SNPs	Unit of MR estimated β	β	SE	*p*	OR	95% CI
EA	Type 2 Diabetes	MR Egger	1208	Log-odds per 4.2 years of schooling	-0.4811	0.1171	4.24E-05	0.62	0.49-0.78
		Weighted Median	1208		-0.5977	0.0329	7.58E-74	0.55	0.51-0.59
		**IVW**	**1208**		**-0.6381**	**0.0334**	**2.74E-81**	**0.53**	**0.49-0.56**

ESM Table 4. Mendelian randomisation analysis of the effect of educational attainment on mediators

Exposure	Outcome	Method	N of SNPs	Unit of MR estimated β	β	SE	*p*	OR	95% CI
EA	BMI	MR Egger	1202	kg/m^2 per 4.2 years of schooling	-0.2947	0.0540	6.04E-08	\	\
		Weighted median	1202		-0.2889	0.0115	3.46E-138	\	\
		**IVW**	**1202**		**-0.3421**	**0.0153**	**5.26E-111**	****	****
EA	SBP	MR Egger	1184	mmHg per 4.2 years of schooling	-1.5282	0.7597	4.45E-02	\	\
		Weighted median	1184		-1.7347	0.1759	6.14E-23	\	\
		**IVW**	**1184**		**-1.8279**	**0.2115**	**5.55E-18**	****	****
EA	DBP	MR Egger	1188	mmHg per 4.2 years of schooling	-0.619	0.4487	1.68E-01	\	\
		Weighted median	1188		-0.8305	0.0966	8.40E-18	\	\
		**IVW**	**1188**		**-0.8127**	**0.1255**	**9.38E-11**	****	****
EA	TV watching	MR Egger	1211	SD (1.5 hours) per 4.2 years of schooling	-0.6218	0.0374	3.76E-56	\	\
		Weighted median	1211		-0.5599	0.0118	5.98E-121	\	\
		**IVW**	**1211**		**-0.6096**	**0.0107**	**4.67E-77**	****	****
EA	Physical activity	MR Egger	1207	SD (undefined) per 4.2 years of schooling	0.0084	0.0630	8.94E-01	\	\
		Weighted median	1207		0.0746	0.0219	6.00E-04	\	\
		**IVW**	**1207**		**0.0809**	**0.0180**	**6.62E-06**	****	****
EA	Smoking	MR Egger	1196	Log-odds per 4.2 years of schooling	-0.415	0.0649	2.33E-10	0.66	0.58- 0.75
		Weighted median	1196		-0.3973	0.0192	4.14E-95	0.67	0.65- 0.70
		**IVW**	**1196**		**-0.4548**	**0.0188**	**7.02E-130**	**0.63**	**0.61- 0.66**

ESM Table 5a. MVMR sensitivity analysis of individual mediating effect

Model	Variable	Method	β	SE	*p*	MVMR Instrument validity test			MVMR Heterogeneity test			MVMR directional pleiotropy test			N of SNPs
						Conditional F-statistics	Q statistics	*p*-value	Q statistic	Q df	*p*-value	Egger intercept	SE	*p*-value	
T2D=EA+SBP	Education	MV-IVW	-0.6010	0.0340	1.23E-62	27.18	3680.70	1.46E-240	3770.50	1237	0.00E+00	-0.001	0.001	3.01E-01	1240
	SBP		0.0186	0.0045	4.05E-05	11.12									
	Edu	MVMR-Egger	-0.4830	0.1190	0.00E+00										
	SBP		0.0190	0.0050	0.00E+00										
T2D=EA+DBP	Education	MV-IVW	-0.6095	0.0335	8.78E-66	27.81	3691.09	4.09E-241	3778.16	1241	0.00E+00	-0.001	0.001	2.82E-01	1244
	DBP		0.0297	0.0077	1.10E-04	10.92									
	Education	MVMR-Egger	-0.4870	0.1190	0.00E+00										
	DBP		0.0300	0.0080	0.00E+00										
T2D=EA+BMI	Education	MV-IVW	-0.2805	0.0349	2.27E-15	24.13	2796.15	5.93E-119	3041.50	1255	0.00E+00	-0.001	0.001	4.22E-01	1258
	BMI		1.0259	0.0560	1.05E-66	22.01									
	Education	MVMR-Egger	-0.2020	0.1040	5.30E-02										
	BMI		1.0250	0.0560	0.00E+00										
T2D=EA+SI	Education	MV-IVW	-0.5616	0.0404	5.43E-41	26.47	3726.57	1.41E-244	3804.01	1248	0.00E+00	-0.001	0.001	3.27E-01	1251
	Smoking		0.1429	0.0507	4.93E-03	2.96									
	Education	MVMR-Egger	-0.4540	0.1170	0.00E+00										
	Smoking		0.1420	0.0510	5.00E-03										
T2D=EA+TV	Education	MV-IVW	-0.2700	0.0622	1.55E-05	23.95	3556.51	6.50E-219	3707.84	1254	0.00E+00	-0.002	0.001	2.43E-01	1257
	TV watching		0.5918	0.0867	1.36E-11	2.47									
	Education	MVMR-Egger	-0.1410	0.1260	2.63E-01										
	TV watching		0.5940	0.0870	0.00E+00										
T2D=EA+PA	Education	MV-IVW	-0.6095	0.0329	7.51E-68	35.17	3569.33	1.25E-221	3762.12	1250	0.00E+00	-0.001	0.001	3.47E-01	1253
	Physical activity		-0.2763	0.0524	1.55E-07	1.82									
	Education	MVMR-Egger	-0.5050	0.1160	0.00E+00										
	Physical activity		-0.2750	0.0520	0.00E+00										

ESM Table 5b. MVMR sensitivity analysis of combined mediating effect after adjusting for two mediators

Model	Variable	Method	β	SE	*p*	MVMR Instrument validity test			MVMR Heterogeneity test			MVMR directional pleiotropy test			N of SNPs
						Conditional F-statistics	Q statistics	*p*-value	Q statistic	Q df	*p*-value	Egger intercept	SE	*p*-value	
T2D=EA+TV+SI	Education	MV-IVW	-0.2053	0.0659	1.89E-03	22.75	3497.33	2.07E-212	3660.26	1246	0.00E+00	-0.001	0.001	2.81E-01	1250
	TV watching		0.5909	0.0869	1.63E-11	2.35									
	Smoking		0.1330	0.0498	7.72E-03	2.97									
	Education	MVMR-Egger	-0.0870	0.1280	4.93E-01										
	TV watching		0.5930	0.0870	0.00E+00										
	Smoking		0.1320	0.0500	8.00E-03										
T2D=EA+TV+PA	Education	MV-IVW	-0.2932	0.0623	2.84E-06	22.67	3460.62	1.28E-206	3657.77	1249	0.00E+00	-0.001	0.001	2.79E-01	1253
	TV watching		0.5243	0.0882	3.54E-09	2.20									
	Physical activity		0.2150	0.0527	4.78E-05	1.70									
	Education	MVMR-Egger	-0.1730	0.1270	1.72E-01										
	TV watching		0.5260	0.0880	0.00E+00										
	Physical activity		0.2130	0.0530	0.00E+00										
T2D=EA+SI+PA	Education	MV-IVW	-0.5441	0.0402	4.72E-39	16.49	3515.09	8.38E-216	3712.57	1242	0.00E+00	-0.001	0.001	4.02E-01	1246
	Smoking		0.1336	0.0503	8.03E-03	2.95									
	Physical activity		-0.2768	0.0523	1.45E-07	1.81									
	Education	MVMR-Egger	-0.4520	0.1170	0.00E+00										
	Smoking		0.1330	0.0500	9.00E-03										
	Physical activity		-0.2750	0.0520	0.00E+00										
T2D=EA+SBP+TV	Education	MV-IVW	-0.2665	0.0628	2.36E-05	21.82	3500.88	7.64E-216	3651.62	1233	0.00E+00	-0.002	0.001	2.55E-01	1237
	SBP		0.0161	0.0045	3.33E-04	10.12									
	TV watching		0.5512	0.0877	4.58E-10	2.36									
	Education	MVMR-Egger	-0.1370	0.1300	2.92E-01										
	SBP		0.0160	0.0040	0.00E+00										
	TV watching		0.5530	0.0880	0.00E+00										
T2D=EA+SBP+SI	Education	MV-IVW	-0.5179	0.0418	2.53E-33	20.10	3607.50	1.28E-232	3714.36	1226	0.00E+00	-0.001	0.001	3.79E-01	1230
	SBP		0.0195	0.0045	1.91E-05	9.20									
	Smoking		0.1674	0.0511	1.09E-03	2.95									
	Education	MVMR-Egger	-0.4180	0.1210	1.00E-03										
	SBP		0.0190	0.0050	0.00E+00										
	Smoking		0.1660	0.0510	1.00E-03										
T2D=EA+SBP+DBP	Education	MV-IVW	-0.6019	0.0340	1.05E-62	21.84	3689.60	4.36E-242	3768.91	1236	0.00E+00	-0.001	0.001	3.05E-01	1240
	SBP		0.0134	0.0085	1.16E-01	9.01									
	DBP		0.0106	0.0145	4.65E-01	8.81									
	Education	MVMR-Egger	-0.4840	0.1190	0.00E+00										
	SBP		0.0130	0.0080	1.14E-01										
	DBP		0.0100	0.0140	4.71E-01										
T2D=EA+SBP+PA	Education	MV-IVW	-0.5786	0.0339	1.08E-58	22.61	3488.86	3.75E-214	3690.97	1233	0.00E+00	-0.001	0.001	3.81E-01	1237
	SBP		0.0180	0.0045	6.17E-05	10.27									
	Physical activity		-0.2696	0.0524	3.15E-07	1.82									
	Education	MVMR-Egger	-0.4790	0.1180	0.00E+00										
	SBP		0.0180	0.0040	0.00E+00										
	Physical activity		-0.2680	0.0520	0.00E+00										
T2D=EA+BMI+TV	Education	MV-IVW	-0.1454	0.0566	1.03E-02	23.05	2783.06	9.84E-118	3017.88	1253	0.00E+00	-0.001	0.001	3.92E-01	1257
	BMI		0.9793	0.0578	3.26E-58	16.23									
	TV watching		0.2472	0.0808	2.28E-03	2.22									
	Education	MVMR-Egger	-0.0610	0.1140	5.96E-01										
	BMI		0.9780	0.0580	0.00E+00										
	TV watching		0.2490	0.0810	2.00E-03										
T2D=EA+BMI+SI	Education	MV-IVW	-0.2627	0.0396	4.61E-11	21.90	2770.84	2.59E-117	3013.56	1247	0.00E+00	-0.001	0.001	4.65E-01	1251
	BMI		1.0255	0.0566	2.99E-65	16.99									
	Smoking		0.0314	0.0456	4.92E-01	2.91									
	Education	MVMR-Egger	-0.1910	0.1060	7.00E-02										
	BMI		1.0250	0.0570	0.00E+00										
	Smoking		0.0310	0.0460	5.03E-01										
T2D=EA+BMI+SBP	Education	MV-IVW	-0.2307	0.0359	1.92E-10	21.58	2659.42	1.59E-106	2934.64	1233	0.00E+00	-0.001	0.001	4.47E-01	1237
	BMI		1.0487	0.0559	3.28E-69	17.16									
	SBP		0.0239	0.0040	2.95E-09	15.21									
	Education	MVMR-Egger	-0.1540	0.1070	1.50E-01										
	BMI		1.0480	0.0560	0.00E+00										
	SBP		0.0240	0.0040	0.00E+00										
T2D=EA+BMI+PA	Education	MV-IVW	-0.2808	0.0350	2.57E-15	23.86	2781.04	3.47E-118	3027.19	1249	0.00E+00	-0.001	0.001	4.24E-01	1253
	BMI		0.9999	0.0574	4.54E-61	16.32									
	Physical activity		-0.1021	0.0480	3.38E-02	1.73									
	Education	MVMR-Egger	-0.2010	0.1050	5.60E-02										
	BMI		0.9990	0.0570	0.00E+00										
	Physical activity		-0.1010	0.0480	3.60E-02										
T2D=EA+BMI+DBP	Education	MV-IVW	-0.2430	0.0356	1.41E-11	22.52	2662.03	5.23E-108	2927.59	1226	0.00E+00	-0.001	0.001	4.89E-01	1230
	BMI		1.0448	0.0562	3.43E-68	13.20									
	DBP		0.0359	0.0069	2.00E-07	9.46									
	Education	MVMR-Egger	-0.1730	0.1070	1.05E-01										
	BMI		1.0440	0.0560	0.00E+00										
	DBP		0.0360	0.0070	0.00E+00										
T2D=EA+BMI+DBP	Education	MV-IVW	-0.2430	0.0356	1.41E-11	22.52	2662.03	5.23E-108	2927.59	1226	0.00E+00	-0.001	0.001	4.89E-01	1230
	BMI		1.0448	0.0562	3.43E-68	13.20									
	DBP		0.0359	0.0069	2.00E-07	9.46									
	Education	MVMR-Egger	-0.1730	0.1070	1.05E-01										
	BMI		1.0440	0.0560	0.00E+00										
	DBP		0.0360	0.0070	0.00E+00										

ESM Table 5c. MVMR sensitivity analysis of combined mediating effect after adjusting for three mediators

Model	Variable	Method	β	SE	*p*	MVMR Instrument validity test			MVMR Heterogeneity test			MVMR directional pleiotropy test			N of SNPs
						Conditional F-statistics	Q statistics	*p*-value	Q statistic	Q df	*p*-value	Egger intercept	SE	*p*-value	
T2D=EA+SI+TV+PA	Education		-0.2313	0.0661	4.84E-04	22.54	3404.42	1.35E-200	3610.14	1241	0.00E+00	-0.001	0.001	3.21E-01	1246
	Smoking	MV-	0.1279	0.0497	1.01E-02	2.95									
	TV watching	IVW	0.5226	0.0884	4.37E-09	2.19									
	Physical activity		-0.2150	0.0527	4.78E-05	1.70									
	Education		-0.1220	0.1280	3.41E-01										
	Smoking	MVMR-	0.1270	0.0500	1.10E-02										
	TV watching	Egger	0.5250	0.0880	0.00E+00										
	Physical activity		-0.2130	0.0530	0.00E+00										
T2D=EA+BMI+TV+PA	Education		-0.1559	0.0571	6.46E-03	22.80	2774.90	1.26E-117	3008.53	1248	0.00E+00	-0.001	0.001	3.83E-01	1253
	BMI	MV-	0.9641	0.0587	4.38E-55	15.69									
	TV watching	IVW	0.2264	0.0820	5.84E-03	2.14									
	Physical activity		-0.0818	0.0485	9.16E-02	1.66									
	Education		-0.0680	0.1150	5.53E-01										
	BMI	MVMR-	0.9630	0.0590	0.00E+00										
	TV watching	Egger	0.2280	0.0820	5.00E-03										
	Physical activity		-0.0810	0.0480	9.70E-02										
T2D=EA+BMI+SI+TV	Education		-0.1265	0.0598	3.46E-02	22.89	2757.14	5.06E-116	2989.95	1245	0.00E+00	-0.001	0.001	4.29E-01	1250
	BMI	MV-	0.9782	0.0585	7.50E-57	15.97									
	Smoking	IVW	0.0323	0.0455	4.77E-01	2.92									
	TV watching		0.2486	0.0812	2.24E-03	2.21									
	Education		-0.0480	0.1150	6.75E-01										
	BMI	MVMR-	0.9770	0.0580	0.00E+00										
	Smoking	Egger	0.0310	0.0450	4.89E-01										
	TV watching		0.2500	0.0810	2.00E-03										
T2D=EA+BMI+SI+PA	Education		-0.2638	0.0396	4.23E-11	17.02	2755.59	1.57E-116	2999.10	1241	0.00E+00	-0.001	0.001	4.66E-01	1246
	BMI	MV-	0.9988	0.0581	1.31E-59	16.05									
	Smoking	IVW	0.0299	0.0457	5.13E-01	2.91									
	Physical activity		-0.1034	0.0481	3.19E-02	1.72									
	Education		-0.1920	0.1070	7.20E-02										
	BMI	MVMR-	0.9980	0.0580	0.00E+00										
	Smoking	Egger	0.0290	0.0460	5.25E-01										
	Physical activity		-0.1020	0.0480	3.30E-02										
T2D=EA+BMI+SBP+TV	Education		-0.1311	0.0567	2.10E-02	23.01	2659.26	1.13E-106	2922.26	1232	0.00E+00	-0.001	0.001	4.14E-01	1237
	BMI	MV-	1.0150	0.0578	8.14E-62	15.84									
	SBP	IVW	0.0230	0.0040	1.43E-08	9.03									
	TV watching		0.1841	0.0812	2.36E-02	2.20									
	Education		-0.0480	0.1160	6.79E-01										
	BMI	MVMR-	1.0140	0.0580	0.00E+00										
	SBP	Egger	0.0230	0.0040	0.00E+00										
	TV watching		0.1860	0.0810	2.20E-02										
T2D=EA+BMI+SBP+SI	Education		-0.2023	0.0407	7.85E-07	16.46	2635.24	4.83E-105	2907.50	1225	0.00E+00	-0.001	0.001	5.00E-01	1230
	BMI	MV-	1.0440	0.0566	2.61E-67	14.81									
	SBP	IVW	0.0240	0.0040	3.42E-09	10.15									
	Smoking		0.0581	0.0456	2.03E-01	2.90									
	Education		-0.1350	0.1080	2.12E-01										
	BMI	MVMR-	1.0430	0.0570	0.00E+00										
	SBP	Egger	0.0240	0.0040	0.00E+00										
	Smoking		0.0570	0.0460	2.10E-01										
T2D=EA+BMI+SBP+PA	Education		-0.2314	0.0359	1.62E-10	21.54	2652.10	7.72E-106	2926.32	1232	0.00E+00	-0.001	0.001	4.75E-01	1237
	BMI	MV-	1.0261	0.0571	3.07E-64	16.19									
	SBP	IVW	0.0237	0.0040	4.48E-09	9.16									
	Physical activity		-0.0903	0.0477	5.89E-02	1.73									
	Education		-0.1590	0.1070	1.36E-01										
	BMI	MVMR-	1.0250	0.0570	0.00E+00										
	SBP	Egger	0.0240	0.0040	0.00E+00										
	Physical activity		-0.0890	0.0480	6.10E-02										
T2D=EA+BMI+SBP+DBP	Education		-0.2275	0.0360	3.75E-10	20.48	2644.30	4.24E-106	2910.54	1225	0.00E+00	-0.001	0.001	4.79E-01	1230
	BMI	MV-	1.0528	0.0561	3.24E-69	14.14									
	SBP	IVW	0.0201	0.0075	7.56E-03	10.91									
	DBP		0.0069	0.0128	5.88E-01	8.89									
	Education		-0.1560	0.1070	1.44E-01										
	BMI	MVMR-	1.0520	0.0560	0.00E+00										
	SBP	Egger	0.0200	0.0080	7.00E-03										
	DBP		0.0070	0.0130	5.92E-01										
T2D=EA+BMI+DBP+SI	Education		-0.2214	0.0403	4.87E-08	16.77	2660.98	4.70E-108	2924.58	1225	0.00E+00	-0.001	0.001	5.11E-01	1230
	BMI	MV-	1.0362	0.0567	3.20E-66	12.84									
	DBP	IVW	0.0365	0.0069	1.36E-07	9.44									
	Smoking		0.0523	0.0457	2.53E-01	2.91									
	Education		-0.1550	0.1080	1.51E-01										
	BMI	MVMR-	1.0360	0.0570	0.00E+00										
	DBP	Egger	0.0360	0.0070	0.00E+00										
	Smoking		0.0510	0.0460	2.61E-01										

ESM Table 5d. MVMR sensitivity analysis of combined mediating effect after adjusting for four to six mediators

Model	Variable	Method	β	SE	*p*	MVMR Instrument validity test			MVMR Heterogeneity test			MVMR directional pleiotropy test			N of SNPs
						Conditional F-statistics	Q statistics	*p*-value	Q statistic	Q df	*p*-value	Egger intercept	SE	*p*-value	
T2D=EA+SBP+SI+TV+PA	Education	MV-IVW	-0.2201	0.0666	9.70E-04	12.58	3341.07	1.41E-195	3552.57	1224	0.00E+00	-0.001	0.001	3.67E-01	1230
	SBP		0.0168	0.0045	1.74E-04	8.11									
	Smoking		0.1505	0.0501	2.71E-03	2.94									
	TV watching		0.4764	0.0891	1.06E-07	2.19									
	Physical activity		-0.2144	0.0528	5.18E-05	1.71									
	Education	MVMR-Egger	-0.1190	0.1310	3.63E-01										
	SBP		0.0170	0.0040	0.00E+00										
	Smoking		0.1490	0.0500	3.00E-03										
	TV watching		0.4790	0.0890	0.00E+00										
	Physical activity		-0.2130	0.0530	0.00E+00										
T2D=EA+BMI+SBP+TV+PA	Education	MV-IVW	-0.1382	0.0573	1.60E-02	12.86	2633.02	5.97E-105	2892.93	1224	0.00E+00	-0.001	0.001	4.66E-01	1230
	BMI		1.0031	0.0588	1.16E-58	10.48									
	SBP		0.0224	0.0040	3.41E-08	7.28									
	TV watching		0.1649	0.0825	4.58E-02	2.11									
	Physical activity		-0.0777	0.0483	1.08E-01	1.67									
	Education	MVMR-Egger	-0.0640	0.1170	5.84E-01										
	BMI		1.0020	0.0590	0.00E+00										
	SBP		0.0220	0.0040	0.00E+00										
	TV watching		0.1670	0.0830	4.30E-02										
	Physical activity		-0.0770	0.0480	1.13E-01										
T2D=EA+BMI+SBP+SI+TV	Education	MV-IVW	-0.1025	0.0600	8.76E-02	12.79	2634.48	4.04E-105	2895.20	1224	0.00E+00	-0.001	0.001	4.62E-01	1230
	BMI		1.0100	0.0584	4.60E-60	10.52									
	SBP		0.0230	0.0040	1.71E-08	7.97									
	Smoking		0.0579	0.0455	2.04E-01	2.90									
	TV watching		0.1846	0.0816	2.38E-02	2.18									
	Education	MVMR-Egger	-0.0280	0.1180	8.10E-01										
	BMI		1.0090	0.0580	0.00E+00										
	SBP		0.0230	0.0040	0.00E+00										
	Smoking		0.0570	0.0460	2.12E-01										
	TV watching		0.1860	0.0820	2.30E-02										
T2D=EA+BMI+SBP+SI+PA	Education	MV-IVW	-0.2034	0.0407	6.63E-07	16.42	2627.73	2.47E-104	2898.93	1224	0.00E+00	-0.001	0.001	5.29E-01	1230
	BMI		1.0206	0.0578	2.43E-62	11.96									
	SBP		0.0237	0.0040	5.19E-09	8.10									
	Smoking		0.0575	0.0456	2.07E-01	2.90									
	Physical activity		-0.0918	0.0478	5.53E-02	1.73									
	Education	MVMR-Egger	-0.1400	0.1080	1.93E-01										
	BMI		1.0200	0.0580	0.00E+00										
	SBP		0.0240	0.0040	0.00E+00										
	Smoking		0.0570	0.0460	2.14E-01										
	Physical activity		-0.0910	0.0480	5.70E-02										
T2D=EA+BMI+SBP+DBP+TV	Education	MV-IVW	-0.1328	0.0569	1.98E-02	12.91	2664.21	2.02E-107	2921.96	1231	0.00E+00	-0.001	0.001	4.17E-01	1237
	BMI		1.0151	0.0578	8.80E-62	11.86									
	SBP		0.0207	0.0075	5.87E-03	8.90									
	DBP		0.1819	0.0815	2.58E-02	7.20									
	TV watching		0.0046	0.0128	7.18E-01	2.18									
	Education	MVMR-Egger	-0.0500	0.1170	6.67E-01										
	BMI		1.0140	0.0580	0.00E+00										
	SBP		0.0210	0.0070	6.00E-03										
	DBP		0.1830	0.0820	2.40E-02										
	TV watching		0.0050	0.0130	7.25E-01										
T2D=EA+BMI+SBP+DBP+SI	Education	MV-IVW	-0.2031	0.0408	7.27E-07	12.04	2642.26	4.98E-106	2906.82	1224	0.00E+00	-0.001	0.001	5.03E-01	1230
	BMI		1.0435	0.0566	3.41E-67	10.81									
	SBP		0.0205	0.0075	6.37E-03	8.90									
	DBP		0.0070	0.0128	5.87E-01	7.88									
	Smoking		0.0581	0.0456	2.03E-01	2.90									
	Education	MVMR-Egger	-0.1360	0.1080	2.08E-01										
	BMI		1.0430	0.0570	0.00E+00										
	SBP		0.0210	0.0080	6.00E-03										
	DBP		0.0070	0.0130	5.91E-01										
	Smoking		0.0570	0.0460	2.10E-01										
T2D=EA+BMI+SBP+DBP+SI+TV	Education	MV-IVW	-0.1043	0.0602	8.33E-02	12.69	2639.50	7.12E-106	2894.89	1223	0.00E+00	-0.001	0.001	4.65E-01	1230
	BMI		1.0101	0.0585	4.99E-60	10.55									
	SBP		0.0206	0.0075	6.03E-03	8.90									
	DBP		0.0047	0.0128	7.12E-01	7.88									
	Smoking		0.0579	0.0456	2.04E-01	2.89									
	TV watching		0.1823	0.0819	2.62E-02	2.17									
	Education	MVMR-Egger	-0.0300	0.1180	7.97E-01										
	BMI		1.0090	0.0580	0.00E+00										
	SBP		0.0210	0.0080	6.00E-03										
	DBP		0.0050	0.0130	7.17E-01										
	Smoking		0.0570	0.0460	2.12E-01										
	TV watching		0.1840	0.0820	2.50E-02										
T2D=EA+BMI+SBP+DBP+SI+PA	Education	MV-IVW	-0.2041	0.0407	6.25E-07	12.02	2633.95	3.17E-105	2898.44	1223	0.00E+00	-0.001	0.001	5.31E-01	1230
	BMI		1.0204	0.0578	2.82E-62	11.98									
	SBP		0.0208	0.0075	5.77E-03	8.90									
	DBP		0.0059	0.0128	6.44E-01	7.88									
	Smoking		0.0575	0.0456	2.07E-01	2.90									
	Physical activity		-0.0908	0.0479	5.82E-02	1.72									
	Education	MVMR-Egger	-0.1410	0.1080	1.91E-01										
	BMI		1.0200	0.0580	0.00E+00										
	SBP		0.0210	0.0080	6.00E-03										
	DBP		0.0060	0.0130	6.47E-01										
	Smoking		0.0570	0.0460	2.14E-01										
	Physical activity		-0.0900	0.0480	6.00E-02										

T2D=EA+BMI+SBP+DBP+SI+TV+PA	Education	MV-IVW	-0.1157	0.0606	5.65E-02	11.60	2634.74	1.74E-105	2889.08	1222	0.00E+00	-0.001	0.001	4.91E-01	1230
	BMI		0.9941	0.0593	5.62E-57	10.30									
	SBP		0.0208	0.0075	5.58E-03	8.90									
	DBP		0.0041	0.0128	7.50E-01	7.79									
	Smoking		0.0574	0.0455	2.08E-01	2.88									
	TV watching		0.1629	0.0827	4.91E-02	2.10									
	Physical activity		-0.0768	0.0484	1.13E-01	1.67									
	Education	MVMR-Egger	-0.0460	0.1180	6.97E-01										
	BMI		0.9930	0.0590	0.00E+00										
	SBP		0.0210	0.0080	5.00E-03										
	DBP		0.0040	0.0130	7.54E-01										
	Smoking		0.0560	0.0460	2.15E-01										
	TV watching		0.1650	0.0830	4.70E-02										
	Physical activity		-0.0760	0.0480	1.17E-01										

ESM Table 6. Reverse MR analysis of mediators to education attainment

Exposure	Outcome	Method	Unit of MR estimated β	MR results			Heterogeneity test		Directional pleiotropy test		N of SNPs				
				β	SE	*p* -value	Q statistic	Q *p*-value	Egger intercept	*p*-value	Original	LD clumping (r2<0.001, 10Mb)	*p*< 5 x10^{-8}	SNPs in outcome	Steiger filtering
BMI	EA	MR Egger	SD (4.2 years of	-0.0257	0.0405	5.26E-01	1888.54	4.46E-232	-0.0017	1.66E-02					
		Weighted median	schooling) per	-0.0844	0.0140	1.79E-09	NA	NA			941	335	304	304	301
		IVW	kg/m^2	**-0.1154**	**0.0161**	**8.63E-13**	1926.35	1.23E-238							
SBP	EA	MR Egger	SD (4.2 years of	0.0009	0.0025	7.20E-01	366.46	4.06E-30	-0.0011	2.45E-01					
		Weighted median	schooling) per	-0.0007	0.0009	4.30E-01	NA	NA			970	396	110	110	110
		IVW	mmHg	**-0.0018**	**0.0009**	**5.90E-02**	371.15	1.42E-30							
DBP	EA	MR Egger	SD (4.2 years of	-0.0015	0.0039	6.95E-01	331.42	7.62E-27	0.0004	6.79E-01					
		Weighted median	schooling) per	0.0001	0.0015	9.39E-01	NA	NA			962	386	102	102	101
		IVW	mmHg	**-0.0001**	**0.0015**	**9.73E-01**	332.00	1.14E-26							
TV watching	EA	MR Egger	SD (4.2 years of	-0.5785	0.1502	2.59E-04	379.77	1.76E-44	0.0012	6.39E-01					
		Weighted median	schooling) per	-0.4130	0.0280	4.04E-49	NA	NA			152	95	95	85	76
		IVW	SD (1.5 hours TV watching)	**-0.5094**	**0.0319**	**2.40E-57**	380.99	2.51E-44							
Smoking	EA	MR Egger	SD (4.2 years of	-0.1857	0.0533	6.54E-04	450.84	4.44E-33	0.0012	2.61E-01					
		Weighted median	schooling) per	-0.1371	0.0146	4.55E-21	NA	NA			378	202	202	200	153
		IVW	Log-odds increase in ever smoking	**-0.1275**	**0.0137**	**1.14E-20**	454.80	2.03E-33							
Physical activity	EA	MR Egger	SD (4.2 years of	NA	NA	NA	NA	NA	NA	NA					
		Weighted median	schooling) per	NA	NA	NA	NA	NA	NA	NA	5	4	4	2	2
		IVW	SD (undefined)	**0.0753**	**0.1160**	**5.16E-01**	6.02	1.41E-02	NA	NA					

ESM Table 7. Characteristics of 1912 Non-Hispanic white participants from the NHANES 2013-2014 survey

Characteristic	
Age	55 [42-70]
Sex	
Male	909 (48%)
Female	1003 (52%)
Body-mass index (kg/m^2)	29.39 ± 6.95
Systolic blood pressure (mmHg)	123.93 ± 18.05
Diastolic blood pressure (mmHg)	69.70 ± 12.14
Television watching	
< 1 hour	216 (11%)
1 hour	277 (14%)
2 hours	442 (23%)
3 hours	349 (18%)
4 hours	287 (15%)
≥5 hours	341 (18%)
Smoking	
Never smoker	914 (48%)
Former smoker	579 (30%)
Current smoker	419 (22%)
HbA_{1c} (%)	5.5 [5.2-5.8]
2h-oral glucose tolerance test (mmol/L)	6.1 [5.1-7.7]
Fasting glucose (mmol/L)	5.6 [5.2-6.1]
Self-reported doctor-diagnosed diabetes	
Yes	242 (13%)
No	1607 (84%)
Borderline	63 (3%)
Taking insulin now	
Yes	80 (4%)
No	1832 (96%)
Taking glucose lowering pills now	
Yes	179 (9%)
No	233 (12%)
Not available	1500 (78%)
Type 2 Diabetes mellitus	
Yes	334 (17%)
No	1578 (83%)
Highest completed educational level	
Less than 9th grade	49 (3%)
9-11th grade (includes 12th grade with no diploma)	210 (11%)
High school graduate/GED or equivalent	431 (23%)
Some college or AA degree	619 (32%)
College graduate or above	603 (32%)
Years of schooling	15 [13-20]

Characteristics of the 1912 non-Hispanic white participants of the NHANES 2013-2014 survey. Data are presented as mean ± standard deviation, median [interquartile range] in case of non-normal distributions, or n (%) in case of categorical variables.

ESM Table 8. Mediation analysis in 1912 Non-Hispanic white participants from the NHANES 2013-2014 survey

Exposure	Mediator	Outcome	a	se_a	b	se_b	c'	se_c'	a*b	se_a*b	c=a*b+c'	Proportion mediated
EA	BMI (kg/m^2)	T2D	-1.218	0.267	0.107	0.012	-0.383	0.074	-0.130	0.032	-0.514	0.253
EA	TV (hours)	T2D	-0.422	0.039	0.176	0.050	-0.381	0.073	-0.074	0.022	-0.455	0.163
EA	Smoking (ever vs never)	T2D	-0.142	0.020	0.229	0.136	-0.419	0.071	-0.033	0.020	-0.451	0.072
EA	SBP (mmHg)	T2D	-1.608	0.518	0.012	0.004	-0.438	0.069	-0.020	0.009	-0.458	0.044
EA	DBP (mmHg)	T2D	-0.102	0.262	-0.008	0.008	-0.445	0.067	0.001	0.002	-0.445	-0.002

Single mediator analysis in NHANES 2013-2014.
a represents an estimate of the association between EA and mediator, per 4.2 years of schooling in a linear regression model
b represents an estimate of the association between mediator and T2D, conditional on EA in a logistic regression model
c' represents an estimate of the direct effect between EA and T2D, conditional on the mediator in a logistic regression model
a*b represents an estimate of the indirect effect of the mediator on T2D
c=a*b+c' represents an estimate of the total effect of EA on T2D
proportion mediated is calculated by a*b / c
standard errors (se) were calculated using the delta method.
Abbreviations: BMI, body-mass index; DBP, diastolic blood pressure; EA, educational attainment; SBP, systolic blood pressure; T2D, type 2 diabetes; TV, television watching.

ESM Figure 1. Directed acyclic graph for 2-step Mendelian randomisation analysis

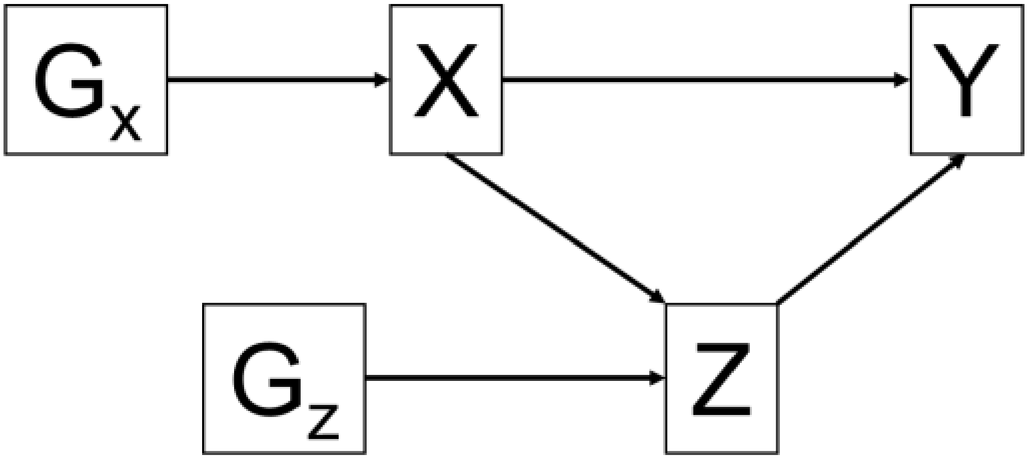

Causal directed acyclic graph illustrating the effect of X on Y through mediation by Z. X is the exposure (education attainment); Y is the outcome (type 2 diabetes) ; Z represents mediators (BMI, SBP, DBP, smoking, TV watching); G_X and G_Z are genetic instruments of X and Z, respectively.

ESM Figure 2. Directed acyclic graph of tested pathways in NHANES 2013-2014

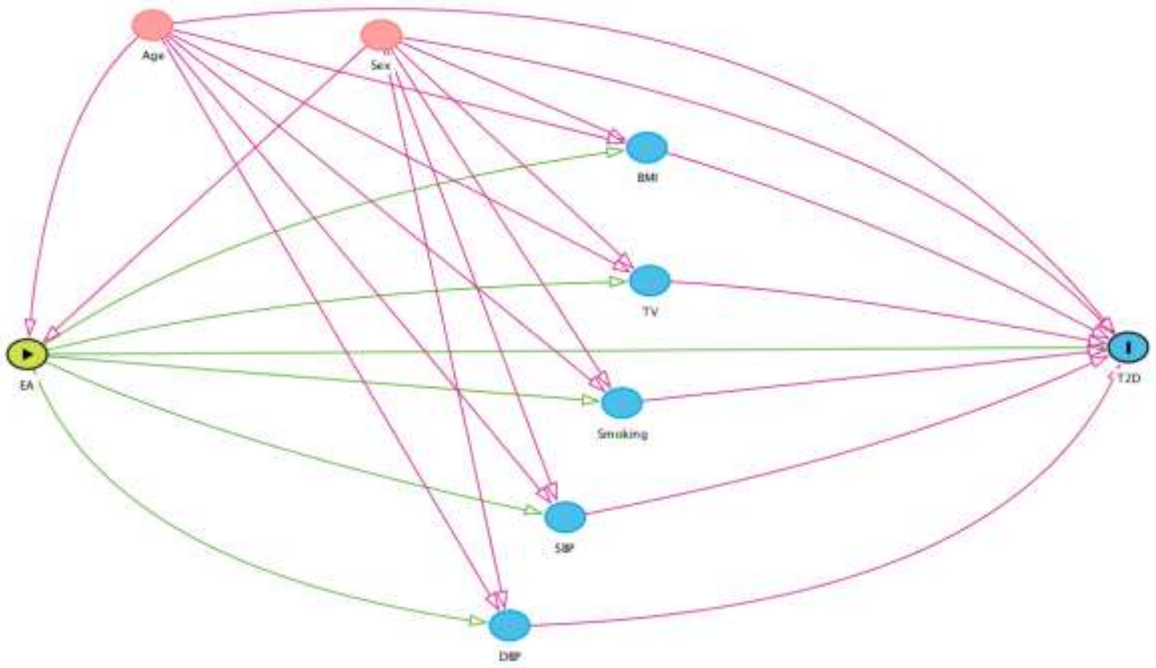

Simplified directed acyclic graph of tested mediation pathways, with mediation by BMI, TV, smoking, SBP, and DBP, whereas age and sex are potential confounders of each relationship. Abbreviations: EA, educational attainment; BMI, body-mass index; TV, television watching; SBP, systolic blood pressure; DBP, diastolic blood pressure; T2D, type 2 diabetes. Drawn using *daggity* software[7].

ESM Figure 3a. Mendelian randomisation scatterplots of education to type 2 diabetes and mediators associations

**A.** Educational attainment > Type 2 diabetes		
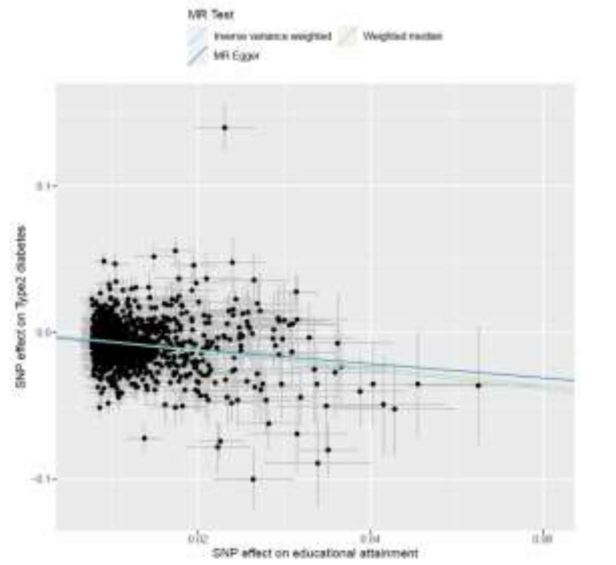		
**B.** Educational attainment > BMI	**C.** Educational attainment > TV watching	**D.** Educational attainment > Smoking
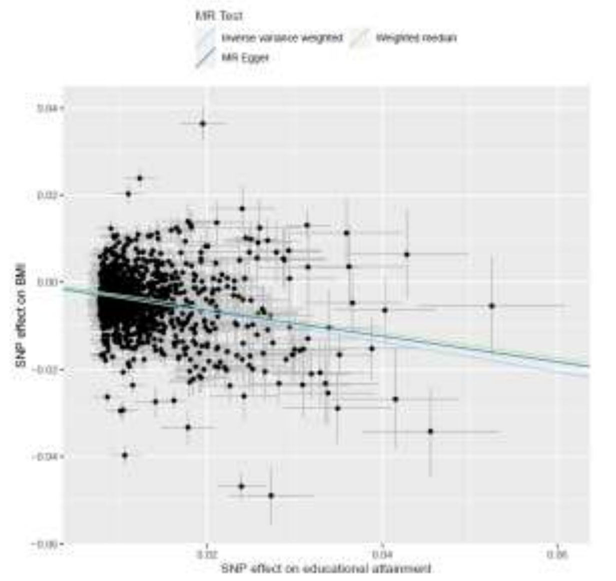	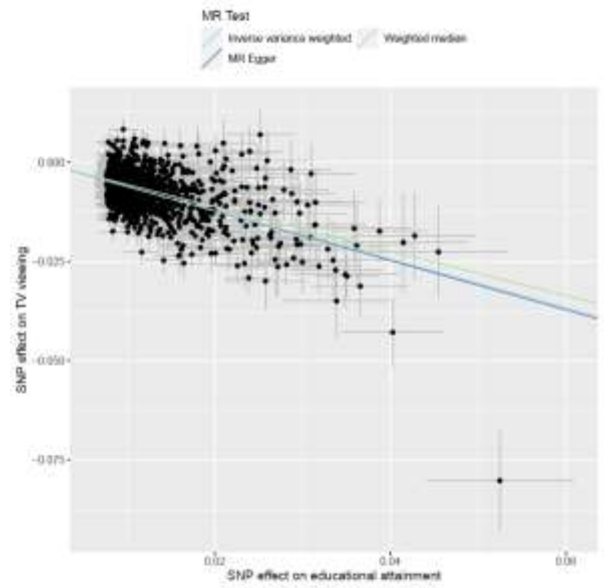	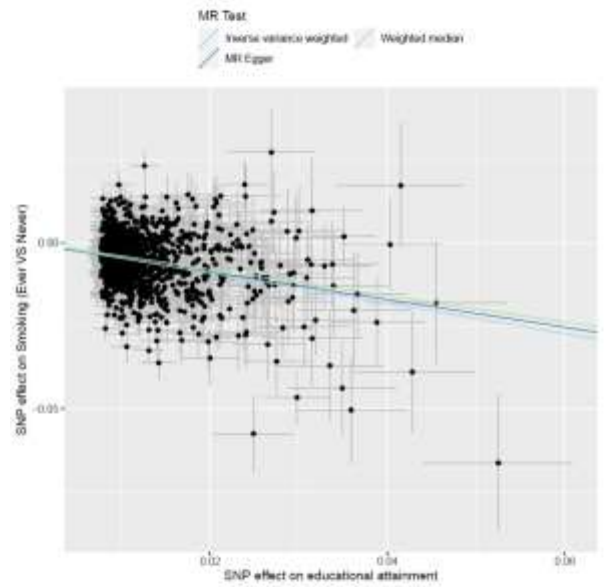
**E.** Educational attainment > Physical activity	**F.** Educational attainment > Systolic blood pressure	**G.** Educational attainment > Diastolic blood pressure
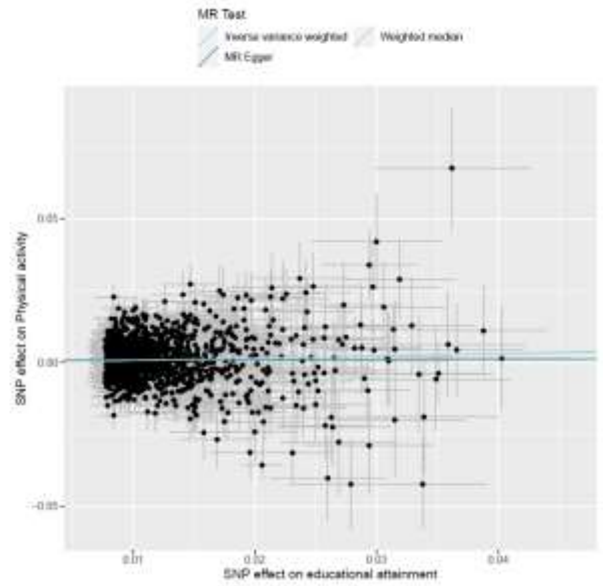	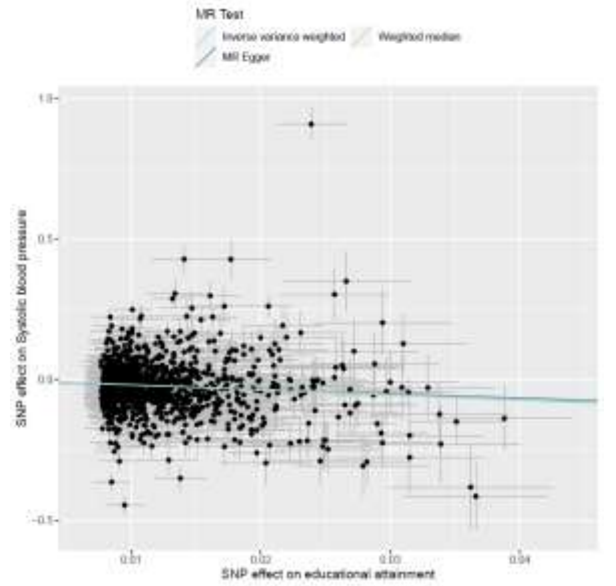	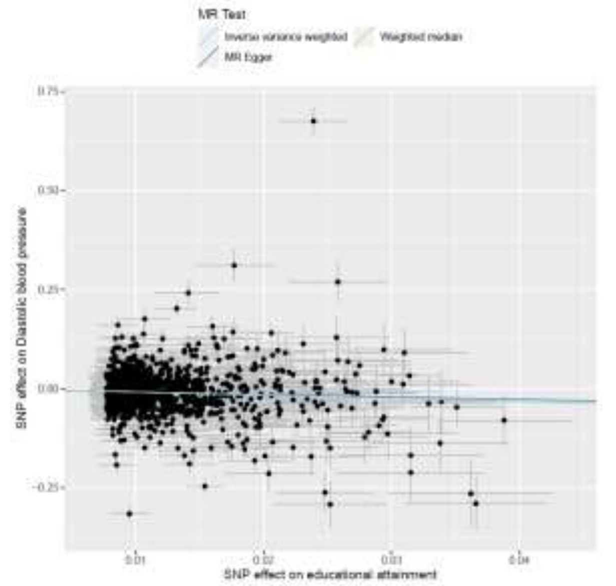

Mendelian randomisation scatterplots. X-axes represent SNP effects on Educational attainment. Y-axes represent SNP effects on (A) Type 2 diabetes, (B) BMI, (C) TV watching, (D) Smoking (ever vs never), (E) Physical activity, (F) Systolic blood pressure, and (G) Diastolic blood pressure. The lines represent pooled estimates of causal effects from inverse variance weighted analysis (lightblue), MR-Egger regression (darkblue), and weighted median analysis (yellow).

ESM Figure 3b. Mendelian randomisation scatterplots of mediators to education associations

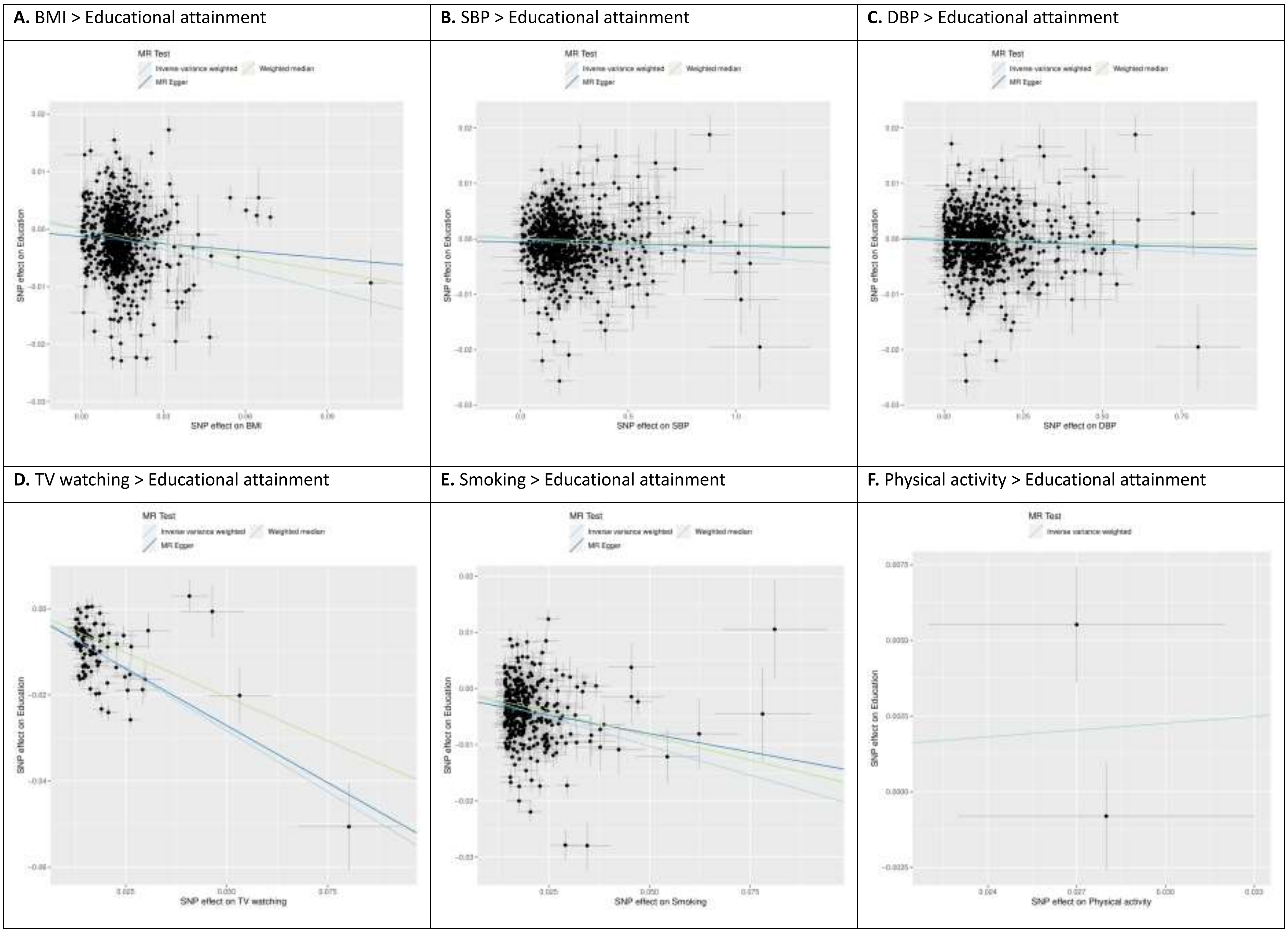

Mendelian randomisation scatterplots. Y-axes represent SNP effects on Educational attainment. X-axes represent SNP effects on (**A**) BMI, (**B**) Systolic blood pressure, (**C**) Diastolic blood pressure, (**D**) TV watching, (**E**) Smoking (ever vs never), and (**F**) Physical activity. The lines represent pooled estimates of causal effects from inverse variance weighted analysis (lightblue), MR-Egger regression (darkblue), and weighted median analysis (yellow).

ESM Figure 4. MVMR estimates of the proportion mediated by each mediator separately using the difference in coefficient method

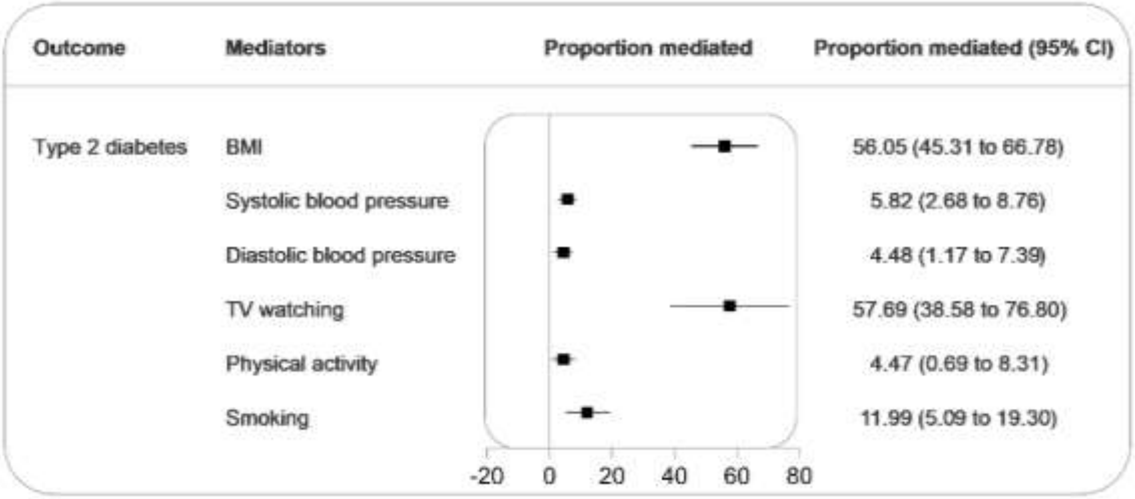

Estimates of individual proportion mediated from multivariable Mendelian randomisation using the difference in regression coefficient method.

ESM Figure 5. Estimates for the effect of education on type 2 diabetes explained by mediators individually (product of coefficients method)

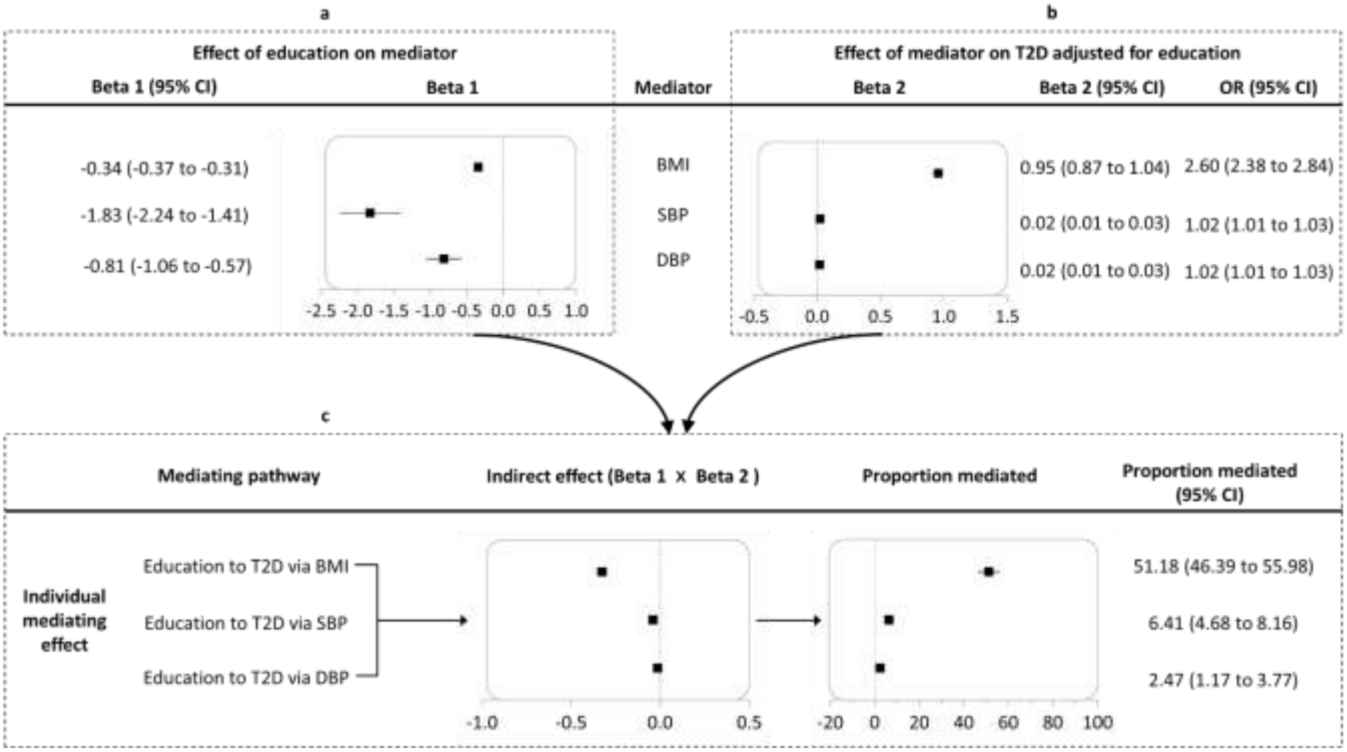

ESM Figure 6. MVMR estimates of combined proportion mediated by selected combinations of mediators

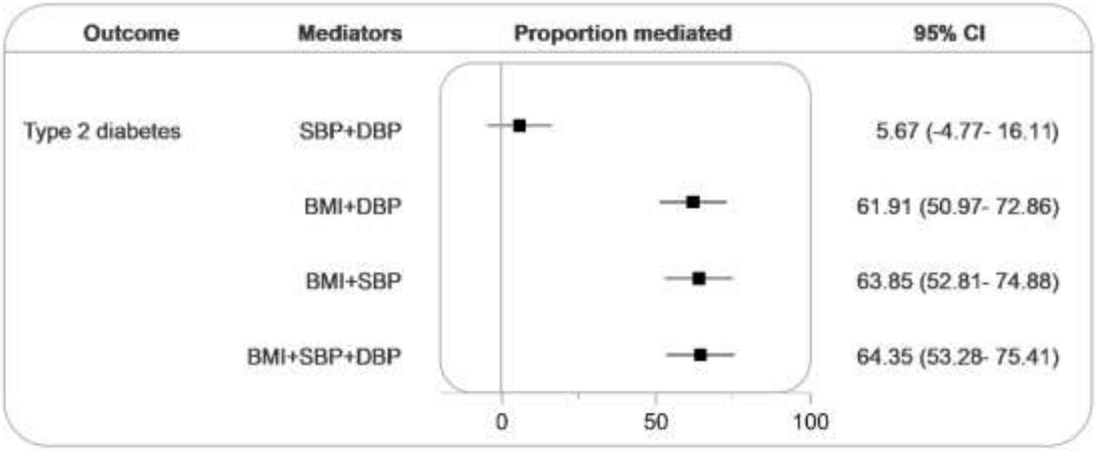

Estimates of combined proportion mediated from multivariable Mendelian randomisation using the difference in regression coefficient method.

Supplementary references

[1] Lawlor DA, Harbord RM, Sterne JA, Timpson N, Davey Smith G (2008) Mendelian randomization: using genes as instruments for making causal inferences in epidemiology. Stat Med 27:1133-1163

[2] Bowden J, Davey Smith G, Burgess S (2015) Mendelian randomization with invalid instruments: effect estimation and bias detection through Egger regression. Int J Epidemiol 44:512-525

[3] Hemani G, Bowden J, Davey Smith G (2018) Evaluating the potential role of pleiotropy in Mendelian randomization studies. Hum Mol Genet 27:R195-R208

[4] Bowden J, Davey Smith G, Haycock PC, Burgess S (2016) Consistent estimation in Mendelian randomization with some invalid instruments using a weighted median estimator. Genet Epidemiol 40:304-314

[5] Lumley T (2004) Analysis of complex survey samples. Journal of statistical software 9:1-19

[6] R Core Team (2014) R: A language and environment for statistical computing. R Foundation for Statistical Computing, Vienna, Austria. URL http://www.R-project.org/.

[7] Textor J, van der Zander B, Gilthorpe MS, Liśkiewicz M, Ellison GT (2016) Robust causal inference using directed acyclic graphs: the R package 'dagitty'. Int J Epidemiol 45:1887-1894
